# microRNA‐125b and its downstream Smurf1/KLF2/ATF2 axis as important promoters on neurological function recovery in rats with spinal cord injury

**DOI:** 10.1111/jcmm.16283

**Published:** 2021-05-05

**Authors:** Kunchi Zhao, Ran Li, Qing Ruan, Chunyang Meng, Fei Yin, Qingsan Zhu

**Affiliations:** ^1^ Department of Spine Surgery China‐Japan Union Hospital of Jilin University Changchun China

**Keywords:** ATF2, KLF2, microRNA‐125b, neurological function recovery, smurf1, spinal cord injury

## Abstract

The purpose of this study is to investigate the role of microRNA‐125b (miR‐125b) and its mechanism in spinal cord injury (SCI) by targeting Smurf1. After loss‐ and gain‐function approaches were conducted in SCI rat models and neural stem cells (NSCs) isolated from foetal rats, the Basso‐Beattie‐Bresnahan (BBB) score was calculated, and related protein expression was determined by Western blot analysis and cell apoptosis by TUNEL staining. NSC viability was detected by CCK‐8, migration abilities by Transwell assay and apoptosis by flow cytometry. The relationship between miR‐125b, Smurf1 and KLF2 was evaluated by dual‐luciferase reporter gene experiments, Co‐IP and in vivo ubiquitin modification assays. Inhibition of miR‐125b and KLF2 and the up‐regulation of Smurf1 and ATF2 were observed in SCI rats. BBB scores were elevated, the expression of Nestin, NeuN, GFAP, NF‐200 and Bcl‐2 protein was enhanced but that of Bax protein was reduced, and cell apoptosis was inhibited in SCI rats after up‐regulating miR‐125b or silencing ATF2. Smurf1 was a target gene of miR‐125b, which promoted KLF2 degradation through its E3 ubiquitin ligase function, and KLF2 repressed the expression of ATF2 in NSCs. The results in vivo were replicated in vitro. miR‐125b overexpression promotes neurological function recovery after SCI.

## 1 INTRODUCTION

Spinal cord injury (SCI) is capable of causing extensive neurological damage on thousands of people every year. It is characterized by non‐penetrating lesions from sports accidents, falls and vehicular, and penetrating bullet wounds and other forms of violence, with an increased propensity for the elderly population.^1^ Epidemiologically speaking, the incidence of SCI in China is at 60 000 per year.^2^ SCI is a kind of traumatic event that affects the physiological, psychological and social welfare of patients,^3^ treatment of which is focused on promoting neurological recovery through alleviating pathophysiological processes after the inciting injury to minimize secondary damage.^4^ It has been documented that cell transplantation is a potential therapy for SCI.^5^ Moreover, despite the improvement of therapies for SCI, there is still no therapeutic approach that specifically targets the neurological deficits caused by SCI.^6^ Hence, it is imperative to explore the mechanism underlying neurological deficits caused by SCI to find out therapies targeting neurological deficits.

Accumulating studies have shown the role of altering levels of microRNAs (miRs) in neurological deficits in humans.^7^ For instance, miR‐125b could repress the neurological deficits in rats with cerebral ischaemia‐reperfusion (CIR) injury.^8^ miR‐125b, being the human orthologue of the miRNA lin‐4, is highly expressed in human cells and tissues, including the brain, thyroid glands and pituitary gland.^9^ Previously reported data have demonstrated that miR‐125b could promote neurological recovery after SCI in rats.^10^ Bioinformatics analysis also confirmed that Smad ubiquitylation regulatory factor‐1 (Smurf1) was targeted by miR‐125b. Smurf1 is a HECT‐type E3 ubiquitin ligase, which has E3 ligase‐dependent and ‐independent activities in a variety of cells.^11^ Thus, Smurf1 has been illustrated to induce neuronal necroptosis after lipopolysaccharide (LPS)‐induced neuroinflammation.^12^ Furthermore, Smurf1 could induce the ubiquitination and degradation of Krüppel‐like factor 2 (KLF2) by serving as an E3 ligase.^13^ KLF2 was reported to exert neuroprotective effects on ischaemic stroke by modulating the blood‐brain barrier function.^14^ KLF2 could down‐regulate the activating transcription factor‐2 (ATF2) to repress the constitutive pro‐inflammatory transcription.^15^ Taken these findings into account, we therefore suggested that miR‐125 may have a pivotal role in SCI via Smurf1/KLF2/ATF2 axis. Thus, we established an SCI rat model and isolated neural stem cells (NSCs) from foetal rats to verify this hypothesis and suggested novel targets for SCI treatment.

## 2 MATERIALS AND METHODS

### 2.1 Compliance with ethical standards

The experiments involving animals followed the principles outlined in the National Institute of Health Guide for the Care and Use of Laboratory animals with the ratification of Animal Experiments in School of Public Health, Jilin University.

### 2.2 Bioinformatics analysis

The downstream target genes of miR‐125b in rats were predicted by the following bioinformatics websites: miRDB (http://www.mirdb.org/), TargetScan (http://www.targetscan.org/vert_72/) and miRWalk (http://mirwalk.umm.uni‐heidelberg.de/). Because the three websites adopted different scoring mechanisms for binding sites, the jvenn tool (http://jvenn.toulouse.inra.fr/app/example.html) was used to obtain the intersection of the three prediction results. The STRING online website (https://string‐db.org/) was employed to conduct interaction analysis of genes to further screen the target genes of miR‐125b, and the analysis results was visualized by the Cytoscape 3.5.1 software. To predict the downstream regulators of genes, the GeneCards database (https://www.genecards.org/) was employed to find the genes that encode for the interaction of proteins and SCI‐related genes. Afterwards, the intersection was obtained by the jvenn tool to screen out the interacting regulatory genes associated with SCI. The coding proteins of genes were classified by Panther website (http://www.pantherdb.org/), and transcription factors were selected for subsequent predictions. To further retrieve the regulatory factors, the co‐expression relationship of transcription factors was searched in the Coexpedia website (http://www.coexpedia.org/). Finally, binding sites of transcription factor on gene promoters were predicted through the GeneCards database.

### 2.3 SCI model in rats

A total of 165 adult male Sprague Dawley (SD) rats (provided by Center of Laboratory Animals, Jilin University, China (license No. SCXK(Ji)2008‐0005)), weighing 180‐220 g, were collected. In addition, 15 rats were grouped as the normal control, 15 rats received sham operation (only removal of T9‐T11 lamina and spinous process), and the remaining rats were adopted for induction of SCI model using the modified Allen method.^16^ Rats were kept in a special cage designated for laboratory rats and were allowed to move freely with access to water and food, prior to the experimental procedures. The rats were anaesthetized by intraperitoneal injection of 20 g/L pentobarbital sodium (40 mg/kg). The posterior midline incision was made to remove the T9 spinous process, part of the T10 spinous process and part of the lamina, with the dura mater exposed. A 10‐g hammer was allowed to fall freely from a height of 25 mm and strike the dural sac with impact energy of 25 mm × 10 g and a damage diameter of 2.5 mm. The bottom of the colliding rod, in contact with the spinal cord, presented an arc‐shaped hollow with a diameter of 2.5 mm, which was identical to the surface of the spinal cord. The successful sign of the strike was the spasmodic swing of the tail, the retracted flapping of the lower limbs and body, and the flaccid paralysis of the lower limbs. After the procedure, the rats were carefully nursed, fed, and the urine was allowed to pass three times a day until the reflex bladder emptying was established. Rats that did not produce the above findings in the modelled group were removed, and the needed rats were supplemented.

### 2.4 Basso‐Beattie‐Bresnahan (BBB) scoring

The motor function of hind limbs of modelled and control rats was observed at different time‐points, that is at day 3, 7, 14, 21 and 28 of the surgical procedure. All rats were allowed to roam freely on a 3‐meterdiameter circular platform, whereas the BBB motor function score was recorded and calculated by two professional trained experimenters. Rats were then placed on the experimental platform to adapt to the environment for 10 minutes, whereas the observation time of each rat was 5 minutes. The experimenters recorded and observed 10 different movements of each rat, including forelimb, elbow joint, trunk, ankle joint, adjacent small joint and tail. The scores recorded by the two different experimenters were averaged and were regarded as the final BBB score of each rat. The score was divided into 3 categories according to the 21 scoring standard: the first category evaluated animals that could not stand (1‐7 points) by assessing the activities of each joint of the hind limbs; the second category evaluated the gait and coordination function of the hind limbs (8‐13 points); the third category evaluated the fine movements of the claws in the movement (14‐21 points).^17^, ^18^

### 2.5 Grouping of SCI rats

The SCI rats were transfected with negative control (NC)‐agomir oligonucleotides, miR‐125b agomir oligonucleotides, overexpression (oe)‐NC lentivirus, short hairpin RNA (sh)‐NC lentivirus, sh‐ATF2 lentivirus, oe‐Smurf1 lentivirus, and oe‐ATF2 lentivirus alone or in combination. After the model was successfully developed, oligonucleotides and lentivirus were diluted in PBS and then intrathecally injected into rats. The rats in each group were protectively injected with 3 μL phosphate buffer saline (PBS) mixture containing 0.5 nmol/L transfection, with strict accordance to the instructions; the rats in the control group were injected with 3 μL PBS for 3 consecutive days.^19^ All oligonucleotides and plasmids were synthesized by Shanghai Genechem Co., Ltd. The PBS was infused into the heart of rats slowly until the limbs and body of rats were completely stiff and straight.

After perfusion, the spinal canal of the rats was carefully cut (without damaging of the spinal cord) and the spinal cord tissues in the injured area were collected and placed in a 10% neutral formalin solution and the number of samples was recorded.

### 2.6 Haematoxylin‐eosin (HE) staining

After the tissues were fixed and dehydrated, they were embedded using a paraffin embedding machine (SPCC‐6D, Dongguan Spectral Standard Experimental Equipment Technology Co., Ltd.) and sectioned into pieces at 2‐4 μmol/L. After conventional dewaxing, the sections were hydrated with gradient alcohol, left to stain with haematoxylin for 5 minutes, differentiated with 1% hydrochloric acid alcohol and blued with 5% ammonia water. The sections were then allowed to stain with 0.5% eosin for 1 minutes and sealed with neutral gum after dehydration and clearing. The pathological changes of the injured spinal cord were observed under a 400 × high power optical microscope. The ratio of inflammatory cell infiltration and necrosis area to the whole visual field area in each visual field was calculated by taking 5 random visual fields from each section: 0 point for no lesion, 1 point for < 25%, 2 points for 25%‐50%, 3 points for 50%‐75% and 4 points for > 75%.

### 2.7 RNA isolation and quantification

The total RNA content in tissue or cell samples was extracted with the TRIzol reagent (Invitrogen) which was pre‐cooled at 4℃. Complementary DNA was synthesized using a PrimerScript reverse transcription Kit or a cDNA reverse transcription Kit (K1622, Beijing Reanta Biotechnology Co., Ltd.). Afterwards, the product was placed in a refrigerator at −80℃ for PCR reaction to occur and to avoid repeated freezing and thawing. Reverse transcription‐quantitative polymerase chain reaction (RT‐qPCR) was operated on an ABI7500 Real‐Time PCR system (Applied Biosystems) using SYBR^®^Premix Ex TaqTM II (Takara). The relative expression level of mRNA or miR was standardized by glyceraldehyde‐3‐phosphate dehydrogenase (GAPDH) or U6 expression. These values were then raised to the power of 2 (2^−ΔΔCt^) to yield fold expression relative to the reference point. The primers are depicted in Table 1.

**TABLE 1:** Primer sequences for RT‐qPCR

Targets	Primer sequence (5'‐3')
miR‐125b	F: TCCCTGAGACCCTAACTTGTGA
	R: GCGAGCACAGAATTAATACGAC
Smurf1	F: GAAACCCAATGGCAGAAA
	R: GCAGATGTTGAGGGATGAG
KLF2	F: GCGGCAAGACCTACACCAAG
	R: GCACAGATGGCACTGGAATG
ATF2	F: GGCAACTTGGGAAGTATTGC
	R: CTCGTTGGTAAAACGCTGGC
U6	F: CTCGCTTCGGCAGCACA
	R: AACGCTTCACGAATTTGCGT
GAPDH	F: GGGAAGGTGAAGGTCGGAGT
	R: TTGAGGTCAATGAAGGGGTCA

### 2.8 Western blot analysis

Radio‐immunoprecipitation assay lysis reagent (RIPA, R0010, Beijing Solarbio Science & Technology Co. Ltd.,) was pre‐cooled down to 4℃ and supplemented with phenylmethylsulphonyl fluoride for the isolation of total protein content from tissues and cells. A bicinchoninic acid protein assay kit (20201ES76, Yeasen Company) was employed for the estimation of protein concentration. After quantification according to different concentrations, the protein was separated by polyacrylamide gel electrophoresis and electroblotted onto a polyvinylidene fluoride membrane (Millipore, Billerica, MA, USA), which was left to seal with 5% bovine serum albumin (BSA) at room temperature for 1 hour. The membrane was subsequently probed overnight at 4°C with the diluted primary antibodies (Abcam) to KLF2 (ab194486, 1:1500, rat), Smurf1 (ab38866, 1:500, rabbit), ATF2 (ab47476, 1:1000, rabbit), Nestin (ab221660, 1:1000, rabbit), NeuN (ab220216, 1:1000, rabbit), glial fibrillary acidic protein (GFAP, ab7260, 1:10 000, rabbit), neurofilament‐200 (NF‐200, ab8135, 1:10 000, rabbit), B‐cell lymphoma‐2 (Bcl‐2, ab59348, 1:10 00, rabbit), Bcl‐2 Associated X Protein (Bax, ab32503, 1:2000, rabbit) and GAPDH (ab181602, 1:10 000, rabbit). The membrane was subsequently incubated with horseradish peroxidase (HRP)‐labelled secondary anti‐rabbit immunoglobulin G (ab6721, 1:10 000, Abcam) at room temperature for 1 hour and was then developed. The grey value of protein bands was analysed by the Quantity One v4.6.2 software. The relative protein expression was expressed by the ratio of the grey value of the corresponding protein bands to that of the GAPDH protein band.

### 2.9 TdT‐mediated dUTP‐biotin nick end‐labelling (TUNEL) staining

The sections were embedded in paraffin, dewaxed and hydrated. The sections were left to immerse in 3% H_2_O_2_ methanol solution for 30 minutes at room temperature to block the activity of peroxidase. Protease K working solution was supplemented to the sections, which were then cultured at 37℃ for 30 minutes. The sections were then placed in 0.1% Triotnx‐100 and 0.1% sodium citrate solutions and recovered to the room temperature for 5 minutes. The sections were supplemented with the freshly prepared TUNEL reaction mixture (prepared according to the instructions of TUNEL Kit), incubated at 37℃ for 90 minutes and recovered to room temperature. The sections were cultured with 5% BSA sealing solution at room temperature in a wet box for 20 minutes, whereas the excess liquid was discarded. The transformed POD solution was supplemented into the sections, and the sections were incubated for 30 minutes at 37℃. Next, the temperature was restored to room temperature. The sections were coloured with diaminobenzidine at room temperature. The reaction time was recorded under a microscope, whereas the reaction was terminated by washing it with water. Five visual fields were obtained from each section to measure the number of TUNEL‐positive cells in each field with 400 or 200 times magnification under a BI‐2000 image analysis system, followed by calculation of the average number of positive cells. Those with brown granules in the nucleus were considered as TUNEL positive cells.

### 2.10 Isolation, culture and identification of NSCs

Foetal SD rats with the gestation age of 14.5‐16.5 days were collected in this study. The foetal rats were aseptically obtained and washed repeatedly with D‐Hanks solution. The skin and muscle tissues were dissected under the guidance of a microscope, and the spinal cord was cut with ophthalmic scissors. The samples were trypsinized and filtered by a 200‐mesh copper mesh, and the supernatant was discarded after centrifugation. The samples were then added with growth medium containing Dulbecco's Modified Eagle Medium (DMEM)/F12, 2% B27, 20 ng/mL basic fibroblast growth factor (bFGF), 20 ng/mL epidermal growth factor (EGF), 100 U/mL penicillin and 100 μg/mL streptomycin, and seeded into a 25 cm^2^ culture flask at the density of 5 × 10^5^ cells/mL, followed by incubation at 37℃ with 5% CO_2_ and saturated humidity. Half of the medium was changed once every 3 days, and cells were passaged once every 7 days. After subsequent subculture, purified cells were obtained. The suspended neurosphere with stable growth at the third passage was harvested for subsequent experiments.

### 2.11 Cell transfection

The cells were seeded onto a 6‐well plate for 24 hours prior to transfection. After achieving approximately 70% confluence, NSCs transfection was conducted with Lipofectamine2000 (11668019, Thermo Fisher Scientific). The NSCs were transfected with the following: NC mimic, miR‐125b mimic, NC inhibitor, miR‐125b inhibitor, oe‐NC plasmid, oe‐Smurf1 plasmid, dimethyl sulphoxide (DMSO), MG132 (proteasome inhibitor), oe‐KLF2 plasmid, and oe‐ATF2 plasmid alone or combinedly. After 6 hours of transfection, the culture medium was renewed to further culture NSCs for 48 hours for the following experiments. Afterwards, RT‐qPCR was performed to detect the silencing efficiency of shRNA (sh‐ATF2#1, sh‐ATF2#2 and sh‐ATF2#3 were set). The shRNA with the lowest expression of ATF2 was selected for subsequent experiments. A specific construction of all plasmids and vectors, sequencing identification, virus packaging and titre detection was conducted by Shanghai Genechem Co., Ltd.

### 2.12 Dual‐luciferase reporter gene assay

We artificially synthesized the target site sequences [wild type (WT)] of human and mouse Smurf1 mRNA 3’untranslated region (UTR) and site‐specific mutated sequence of WT target site [mutant type (MUT)]. Restriction endonuclease was adopted to cleave pmiR‐RB‐REPORTTM plasmids (all constructed by Guangzhou RiboBio Co., Ltd.), to allow WT and MUT segments to be inserted. The correctly sequenced luciferase reporter plasmids, WT and MUT, were employed for the subsequent transfection. Vectors containing MUT and WT were co‐transferred into HEK293T cells (CBP60439, Cobioer Biosciences CO., LTD.) with NC mimic or miR‐125b mimic. After 48 hours, cells were lysed and centrifuged for 3‐5 minutes, followed by the collection of the supernatant. Renilla luciferase assay kit (YDJ2714, Shanghai Yuduo Biotechnology Co., Ltd.,) was employed to determine relative light units (RLU) of luciferase activity. Luciferase activity was analysed on a Dual‐Luciferase Reporter Assay System (Promega Co., Ltd.,) with renilla luciferase activity as an internal reference. Relative luciferase activity was calculated by the ratio of RLU value determined by the firefly luciferase Kit to that determined by Renilla luciferase Kit.

### 2.13 Co‐immunoprecipitation (CO‐IP)

The cells were washed twice with pre‐cooled PBS to remove the residual medium and serum. Following the complete absorbance of PBS, 1 mL of fresh PBS was added and the cells were transferred into a 1.5 mL centrifuge tube and centrifuged for 5 min at 37℃ and 3000 r/min. The supernatant was subsequently discarded. A Protease inhibitor cocktail (Roche Diagnostics GmbH) was supplemented into the E1A lysis buffer, [250 mmol/L NaCl, 50 mmol/L 4‐(2‐hydroxyethyl)‐1‐piperazineëthanesulphonic acid (HEPES; PH 7.5) and 0.1% NP‐40, 5 mmol/L ethylene diamine tetra‐acetic acid (EDTA)] which was lysed by a lysis buffer on ice and sonicated with 3% power for 3 minutes, followed by 15‐min centrifugation at 4℃ and 12 000 r/min. Following that, 40 μL of lysate was taken as the control of IB whereas the remaining 650 μL lysate was added with antibodies and mixed for 3 hours at 4℃. The samples were supplemented with 40 μL of protein A/G‐Agarose, mixed at 4℃ for more than 8 h and washed with 1 mL of E1A lysis for 10 minutes for 3 times. Furthermore, the samples supplemented with E1A lysis and 2 × sample buffer solutions were boiled at 100℃ for 15 minutes. The samples were then subjected to Western blot analysis with the same antibodies used above.

### 2.14 Detection of ubiquitination modification in vivo

When the cell density was approximately 80%, the cells were transfected with various plasmids using the VigoFect transfection reagent and were harvested after 36‐48 hours. RIPA lysis [50 mmol/L Tris‐HCl (PH 7.5), 150 mmol/L NaCl, 5 mmol/L EDTA, 1% (v/v) NP‐40, 1% sodium deoxycholate, 0.1% sodium dodecyl sulphate, protease inhibitor] was applied to lyse the cells that were disrupted by ultrasound. The expression of various plasmids was evaluated in the partial lysate. The rest of the lysate was added with the corresponding antibodies for immunoprecipitation, rotated for 3 hours at 4℃ and mixed with protein A/G‐Agarose for more than 8 hours at 4℃. The cells were then washed at least three times with RIPA lysis and allowed to denature with 2 × sample buffer at 100℃ for 15 minutes. Thereafter, the cells were tested by Western blot analysis (the same antibodies used above) or stored at −20℃.

### 2.15 Cell counting kit (CCK)‐8 assay

The CCK‐8 Kit (CA1210‐100, Solarbio) was employed for cell counting. The cells at the logarithmic growth phase were cultured in a 96‐well plate with 5 × 10^3^ cells/well for 3 days. The cells were detected from 0 hour after seeding. Afterwards, the cells in each well were cultured with 10 μL of CCK‐8 solution for 2 hours. Then, the optical density (OD) value at 450 nm was measured by a microplate reader (Bio‐Rad 680, Bio‐Rad, Hercules) at 24 hours, 48 hours, 72 hours and 96 hours, respectively, followed by plotting of the cell proliferation curve.

### 2.16 Transwell assay

Pre‐cooled Matrigel (40111ES08, Yeasen Company), diluted by serum‐free DMEM (Matrigel: DMEM = 1:2), was placed in the apical chamber of the Transwell chamber (3413, Beijing UNIQUE Biotechnology Co., Ltd., Beijing, China) and incubated at 37℃ for 4‐5 hours to allow coagulation. The transfected cells were then diluted with 100 μL serum‐free medium to prepare the cell suspension, with a concentration of about 1 × 10^6^ cells/mL. The cells were seeded into the apical chamber. The basolateral chamber was added with 500 μL of DMEM containing 20% foetal bovine serum, and each group was set with 3 duplicated wells. After 24‐hours of culture at 37℃ with 5% CO_2_, the Transwell chamber was fixed with 5% glutaraldehyde and was left to stain with 0.1% crystal violet for 5 minutes at 4℃. The cells on the surface were wiped off with cotton balls and observed under an inverted fluorescence microscope (TE2000, Nikon) with 5 visual fields randomly read and photographed. The average number of cells passing through the chamber in each group was calculated.

### 2.17 Flow cytometry

After cells were transfected for 48‐hours, they were treated with 0.25% trypsin without EDTA, collected into a centrifuge tube, centrifuged and followed by the removal of supernatant. After washing, the supernatant was discarded by centrifugation. Annexin‐V/PI staining solution was prepared by mixing Annexin‐V‐FITC, PI and HEPES buffer at the ratio of 1:2: 50, according to the instructions of Annexin V‐fluorescein isothiocyanate (FITC)/propidium iodide (PI) double staining kit (556547, Shanghai Solja Technology Co. Ltd.,). Cells at a density of 1 × 10^6^ were suspended by 100 μL staining solution, shaken and mixed well followed by being incubated at room temperature for 15 minutes. Afterwards, one ml of HEPES buffer was added into cells which were then shaken and mixed completely. A flow cytometer (Bio‐Rad ZE5, Bio‐Rad) was adopted to determine cell apoptosis. The maximum absorption wavelength of FITC was at 488 nm, and the excitation wavelength was at 525 nm. The maximum absorption and emission wavelength of the PI‐DNA complex were at 535 nm and 615 nm, respectively.

### 2.18 Statistical analysis

All measurement data were depicted as mean ± standard deviation and analysed by SPSS 21.0 software (IBM) with *P* < .05 as a level of statistical significance. Data between the two groups were compared by an unpaired *t* test, and comparisons among multiple groups were performed with one‐way analysis of variance (ANOVA) whereas pairwise comparison within groups was conducted by post hoc test. Cell viability at different time‐points was compared using two‐way ANOVA, and scores at different time‐points were compared by repeated‐measures ANOVA, followed by Bonferroni post hoc test.

## 3 RESULTS

### 3.1 Overexpression of miR‐125b inhibited SCI and promoted the recovery of nerve function in rats

**FIGURE 1:**
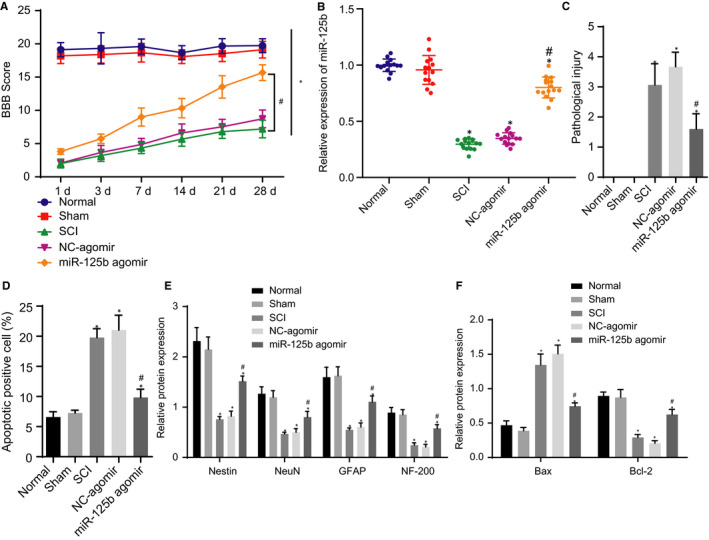
SCI development is repressed and promoted the recovery of nerve function by overexpressing miR‐125b in rats. Normal rats and sham‐operated rats were used as controls, and SCI rats were treated or not treated with NC‐agomir and miR‐125b. A, BBB scores of rats. B, RT‐qPCR detection of miR‐125b expression in the injured spinal cord of rats normalized to U6. C. HE staining analysis of the pathological changes of the spinal cord of rats. D. Apoptosis cells in the spinal cord of rats detected by TUNEL staining. E, Western blot analysis of protein expression of Nestin, NeuN, GFAP and NF‐200 normalized to GAPDH. F, Western blot analysis of expression of Bax and Bcl‐2 protein normalized to GAPDH. ^*^ *P* < .05 vs sham‐operated rats and normal rats, ^#^ *P* < .05 vs SCI rats treated with NC‐agomir. Data between the two groups were compared by an unpaired *t* test, and comparisons among multiple groups were performed with one‐way ANOVA. Scores at different time‐points were compared by repeated measures ANOVA. n = 15 in each group

SCI models were initially generated on SD rats, and the BBB score was utilized to evaluate the motor function of rats. As depicted in Figure 1A, the BBB score of SCI rats was decreased compared to that of normal rats and sham‐operated rats, which suggested the successful establishment of the SCI rat model. On the other hand, RT‐qPCR results showed that the expression of miR‐125b was lowered in SCI rats than that in normal rats and sham‐operated rats (Figure 1B). HE staining revealed that SCI rats showed severe deformation of spinal cord, disorder of tissue structure, unclear boundary of grey‐white matter, disappearance of Nissl body, cell swelling, vacuole degeneration, pyknosis, fragmentation and dissolution of some nerve nuclei, inflammatory cell infiltration, phagocyte aggregation, glial cell proliferation and formation of scar tissue, and vacuole in damage zone, compared with normal rats and sham‐operated rats, which indicated the significant increase in degree of pathological injury (Figure 1C). According to our TUNEL assay results, apoptosis was considered positive when the nuclei were stained in brown. In contrast to normal rats and sham‐operated rats, apoptosis cells in SCI rats were significantly increased (*P* < .05; Figure 1D). Western blot analysis was performed to detect the protein expressions of neural stem cell marker factor (Nestin), neuron factor (NeuN), GFAP, NF‐200 and apoptosis‐related factors (Bax and Bcl‐2). The expression of Nestin, NeuN, GFAP, NF‐200 and Bcl‐2 proteins was remarkably lowered, whereas the expression of Bax protein was higher in SCI rats than that in normal rats and sham‐operated rats (*P* < .05; Figure 1E,F), indicating that the proliferation and differentiation of neurons were inhibited whereas apoptosis was increased. In conclusion, we found that miR‐125b had a lower expression in SCI rats.

Furthermore, transfected NC‐agomir and miR‐125b agomir oligonucleotides were transfected into the SCI modelled rats. The BBB scores were calculated and demonstrated that the BBB score of miR‐125b agomir‐treated SCI rats was significantly elevated (Figure 1A). RT‐qPCR results indicated that the expression of miR‐125b was enhanced in SCI rats with miR‐125b agomir (Figure 1B). Moreover, HE staining showed that the pathological injury degree of SCI rats was decreased with miR‐125b agomir (Figure 1C). TUNEL assay results documented that treatment with miR‐125b agomir potently reduced apoptotic cells in SCI rats (Figure 1D). The results of Western blot analysis in SCI rats revealed that the expression of Nestin, NeuN, GFAP, NF‐200 and Bcl‐2 protein was significantly increased; however, the expression of Bax protein was lowered after treatment with miR‐125b agomir (Figure 1E,F). Therefore, our findings revealed that the overexpression of miR‐125b could prevent the pathological process of spinal cord injury, promote the expression of NSC‐ and neuron cell‐related factors, inhibit their apoptosis and restore neural function.

### 3.2 miR‐125b targeted down‐regulated Smurf1

**FIGURE 2:**
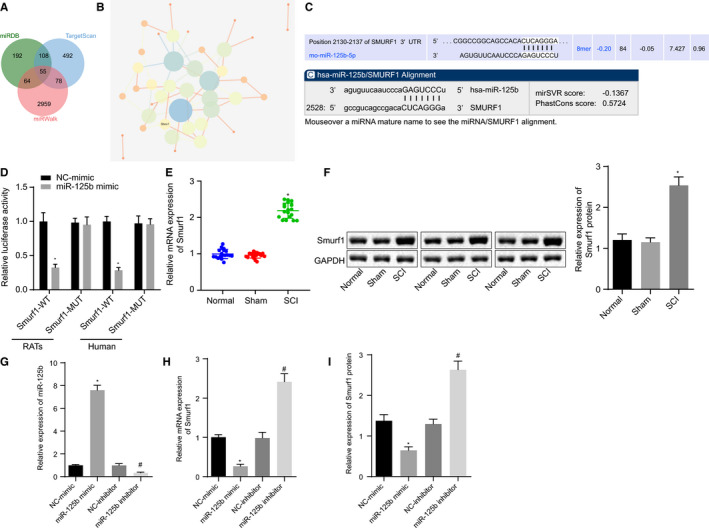
Smurf1 is a target gene of miR‐125b in NSCs. A, A Venn diagram of the intersection of the predicted downstream target genes of miR‐125b in miRDB, TargetScan and miRWalk bioinformatics websites. B, The network of interaction relationship among 55 candidate genes analysed by STRING website. The circles in the figure from large to small represent degree values of genes from large to small, the circle colours from blue to orange represent Degree from large to small, and the lines in the middle of the circles represent co‐expression relationships among genes. C, Binding site between miR‐125b and Smurf1 in rats and human predicted by Targetscan and miRanda websites. D, The targeting relationship between miR‐125b and Smurf1 verified by dual‐luciferase reporter gene assay. E, RT‐qPCR detection of the expression of Smurf1 in the spinal cord of rats after modelling normalized to GAPDH. F, Western blot analysis of the expression of Smurf1 protein in the spinal cord after modelling normalized to GAPDH. NSCs were transfected with miR‐125b mimic, NC mimic, miR‐125b inhibitor and NC inhibitor. G, Expression of miR‐125b in NSCs detected by RT‐qPCR normalized to U6. H, Expression of Smurf1 mRNA in NSCs detected by RT‐qPCR normalized to GAPDH. I, Expression of Smurf1 protein in NSCs detected by Western blot analysis normalized to GAPDH. ^*^ *P* < .05 vs sham‐operated rats and normal rats, or the transfection with NC mimic, ^#^ *P* < .05 vs NSCs transfected with NC inhibitor. Data between the two groups were compared by an unpaired *t* test and comparisons among multiple groups were performed with one‐way ANOVA. Rats: n = 15 in each group. The cell experiment was repeated three times

Following the identification on the critical role of miR‐125b in SCI, we then aimed to explore the downstream mechanism of miR‐125b in SCI. Bioinformatics websites including miRDB, TargetScan and miRWalk were utilized to predict the downstream target genes of miR‐125b, and 55 genes were collected from the intersection of the three prediction results (Figure 2A). After further analysis of gene interaction through the STRING online website and visualization of analysis results using Cytoscape 3.5.1 software, it was found that Smurf1 gene was at the core of the interaction network (Figure 2B). Our data from the Targetscan and miRanda websites illustrated that the miR‐125b and Smurf1 3'UTR in rats and human had a targeted binding site (Figure 2C). Dual‐luciferase reporter gene assay depicted that the luciferase activity of Smurf1‐WT was significantly decreased after the transfection of miR‐125b mimic (*P* < .05), whereas smurf1‐MUT exhibited no significant difference (*P* > .05), indicating that Smurf1 was the target gene of miR‐125b (Figure 2D). Furthermore, RT‐qPCR and Western blot analysis showed that the mRNA (Figure 2E) and protein (Figure 2F) expression of Smurf1 in SCI rats was significantly increased, compared with that of normal rats and sham‐operated rats (*P* < .05).

Furthermore, NSCs were selected for the subsequent experiments because of their high differentiation and migration abilities. NSCs are notably characterized for their continuous differentiation into neurons in the process of normal neuroregulation, to supplement dead or damaged neurons to maintain the normal process of neuroregulation. Thus, NSCs were later transfected with miR‐125b mimic, miR‐125b inhibitor and their NCs. As shown in the results of Figure 2G‐I, the expression of miR‐125b was enhanced whereas the expression of Smurf1 declined in miR‐125b mimic‐transfected NSCs, which was contrary to NSCs transfected with miR‐125b inhibitor. Our data showed that Smurf1 was highly expressed in SCI tissues, whereas miR‐125b targeted and inhibited the Smurf1 in NSCs.

### 3.3 Overexpression of miR‐125b targeted Smurf1 to promote proliferation and migration but inhibit apoptosis in NSC

**FIGURE 3:**
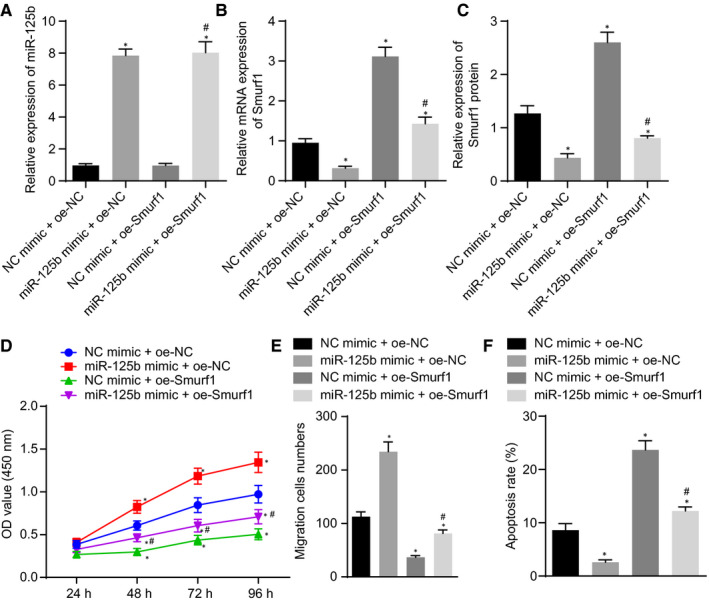
The overexpression of miR‐125b or silencing of Smurf1 induces proliferation and migration but represses apoptosis in NSCs. NSCs were transfected with NC mimic + oe‐NC, miR‐125b mimic + oe‐NC, NC mimic + oe‐Smurf1 or miR‐125b mimic + oe‐Smurf1. A, Expression of miR‐125b in NSCs measured by RT‐qPCR normalized to U6. B, Smurf1 mRNA expression in NSCs measured by RT‐qPCR normalized to GAPDH. C, Expression of Smurf1 protein in NSCs detected by Western blot analysis normalized to GAPDH. D, NSC viability measured by CCK‐8. E, NSC migration evaluated by Transwell assay. F, NSC apoptosis assessed by flow cytometry. ^*^ *P* < .05 vs NSCs transfected with NC mimic + oe‐NC, ^#^ *P* < .05 vs NSCs transfected with NC mimic + oe‐Smurf1. Comparisons among multiple groups were performed with one‐way analysis of variance (ANOVA), and cell viability at different time‐points was compared using two‐way ANOVA. The cell experiment was repeated three times

To further investigate the effect of miR‐125b on the proliferation, migration and apoptosis of NSCs by regulating Smurf1, NSCs were transfected with miR‐125b mimic and oe‐Smurf1. The results of RT‐qPCR and Western blot analysis showed that the expression of miR‐125b in NSCs transfected with miR‐125b mimic was evidently increased, whereas that of Smurf1 was down‐regulated. Further investigation revealed that oe‐Smurf1 treatment did not alter the expression of miR‐125b; however, the expression of Smurf1 was up‐regulated in NSCs. NSCs transfected with NC mimic + oe‐Smurf1 exhibited significantly elevated expression of miR‐125b in comparison with NSCs transfected with miR‐125b mimic + oe‐Smurf1, illustrating the lowered expression of Smurf1 (Figure 3A‐C). CCK‐8 (Figure 3D), Transwell assay (Figure 3E) and flow cytometry (Figure 3F) revealed that miR‐125b mimic enhanced the viability and migration abilities of NSCs although significantly reducing apoptosis in NSCs; however, the outcome was reversed after Smurf1 was overexpressed. In addition, the effects of oe‐Smurf1 on NSC viability, migration and apoptosis were negated by the additional treatment with miR‐125b mimic. Hence, these results demonstrated that miR‐125b could induce proliferation and migration, although repressing the apoptosis in NSCs by targeting Smurf1.

### 3.4 Smurf1 promoted the degradation of KLF2 through its E3 ubiquitin ligase function

**FIGURE 4:**
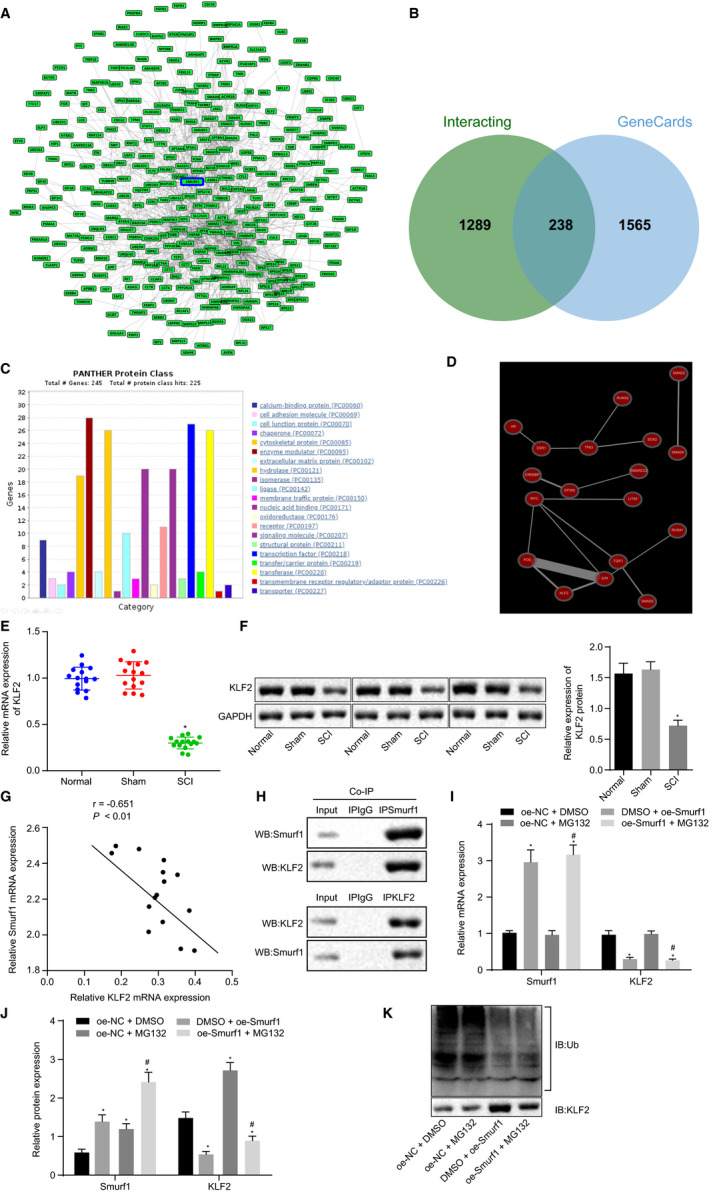
Smurf1 degrades KLF2 via its E3 ubiquitin ligase function. A, Interacting proteins of Smurf1 gene in GeneCards database. B, Venn diagram of intersection of interacting proteins of Smurf1 gene in GeneCards database and SCI‐related genes. C. Classification of coding proteins of genes by Panther website. D, Co‐expression network of transcription factors in Coexpedia database. E, The expression of KLF2 mRNA in the spinal cord of rats after modelling detected by RT‐qPCR normalized to GAPDH. F, The expression of KLF2 protein in the spinal cord of rats after modelling detected by Western blot analysis normalized to GAPDH. G, The correlation between the expression of KLF2 and Smurf1 in SCI rats analysed by Pearson's correlation analysis (n = 15). H, The interaction between Smurf1 and KLF2 verified by the Co‐IP experiment. I, mRNA expression of Smurf1 and KLF2 in the transfected cells detected by RT‐qPCR normalized to GAPDH. J, The expressions of Smurf1 and KLF2 protein in transfected cells detected by Western blot analysis normalized to GAPDH. K, The transfer of Ub molecule by KLF2 in transfected cells detected by Western blot analysis normalized to GAPDH. ^*^ *P* < .05 vs sham‐operated rats and normal rats, or the treatment with oe‐NC + DMSO, ^#^ *P* < .05 vs treatment with oe‐NC + MG132. Comparisons among multiple groups were performed with one‐way ANOVA. Pearson's correlation analysis was conducted for the relationship between KLF2 and Smurf1. Rats: n = 15 in each group. The cell experiments were repeated three times

We aimed to explore the effects of KLF2 on the expression of Smurf1 during SCI. For this purpose, we used the GeneCards database to find 1527 interacting proteins with Smurf1 (Figure 4A) and 1803 genes related to SCI; 238 interacting genes related to SCI were obtained from the intersection (Figure 4B). Twenty‐seven transcription factors were identified by classifying the encoded proteins of the genes on the Panther website (Figure 4C). We then obtained a co‐expression network of transcription factors through the Coexpedia database, with KLF2 at a key position (Figure 4D). RT‐qPCR and Western blot analysis were conducted to detect the expression of KLF2 in SCI rats. Our results demonstrated that the expression of KLF2 in SCI rats was significantly lowered compared with that of normal rats and sham‐operated rats (*P* < .05; Figure 4E,F). Pearson's correlation analysis depicted a negative correlation between the expression of KLF2 and Smurf1 in SCI rats (n = 15) (*P* < .05; Figure 4G). Intriguingly, the results of Co‐IP experiments suggested that overexpressed IP Smurf1 was specifically bound to KLF2 protein, whereas overexpressed IP KLF2 was specifically bound to the Smurf1 protein, indicating that Smurf1 and KLF2 could interact with each other (Figure 4H). Next, we overexpressed Smurf1 in NSCs and added either the proteasome inhibitor MG132 or DMSO. According to Figure 4I,J, Smurf1 mRNA and protein expression was noticeably increased; however, KLF2 was markedly lowered in NSCs treated with DMSO + oe‐Smurf1 (*P* < .05) compared with that of NSCs treated with oe‐NC + DMSO; there was no significant difference in the expression of Smurf1 and KLF2 mRNA in NSCs treated with oe‐NC + MG132 (*P* > .05), whereas the expression of Smurf1 and KLF2 protein was markedly enhanced and the level of KLF2 protein was more significantly elevated (*P* < .05); the expression of Smurf1 mRNA and protein was remarkably increased in NSCs treated with oe‐Smurf1 + MG132, whereas that of KLF2 mRNA was decreased but its protein level was increased (*P* < .05). In contrast to NSCs treated with oe‐NC + MG132, the expression of Smurf1 mRNA and protein in NSCs treated with oe‐Smurf1 + MG132 was significantly elevated, whereas the expression of KLF2 mRNA and protein was decreased (*P* < .05). Moreover, NSCs with overexpressed levels of Smurf1 exhibited a large number of Ub molecules that were transferred by KLF2, thus producing a classical ubiquitin UB molecular distribution band; however, in cells treated with MG132, there were no Ub molecules transferred by KLF2 (*P* < .05; Figure 4K), indicating that Smurf1 promoted the degradation of KLF2 through its E3 ubiquitin ligase function. In summary, our data suggested that the expression of KLF2 in SCI rats was lowered, whereas Smurf1 could promote the degradation of KLF2 through its E3 ubiquitin ligase function.

### 3.5 Smurf1 inhibited proliferation and migration and promoted apoptosis of NSCs by promoting the degradation of KLF2

**FIGURE 5:**
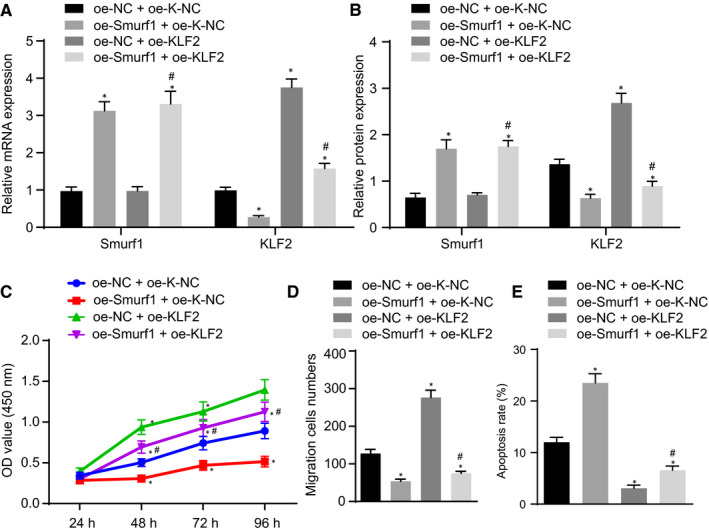
The overexpression of Smurf1 promotes KLF2 degradation to induce apoptosis, but represses proliferation and migration in NSCs. NSCs were transfected with oe‐NC + oe‐K‐NC, oe‐Smurf1 + oe‐K‐NC, oe‐NC + oe‐KLF2 and oe‐Smurf1 + oe‐KLF2. A, The mRNA expression of Smurf1 and KLF2 in NSCs measured by RT‐qPCR normalized to GAPDH. B, Expressions of Smurf1 and KLF2 protein in NSCs detected by Western blot analysis normalized to GAPDH. C, NSC viability measured by CCK‐8. D, NSC migration evaluated by Transwell assay. E, NSC apoptosis assessed by flow cytometry. ^*^ *P* < .05 vs NSCs transfected with oe‐NC + oe‐K‐NC, ^#^ *P* < .05 vs NSCs transfected with oe‐NC + oe‐KLF2. Comparisons among multiple groups were performed with one‐way analysis of variance (ANOVA) and cell viability at different time‐points was compared using two‐way ANOVA. The cell experiment was repeated three times

KLF2 and Smurf1 were overexpressed in NSCs to investigate whether Smurf1 could promote the degradation of KLF2 to regulate the proliferation, migration and apoptosis abilities of NSCs. Our results from the RT‐qPCR and Western blot analysis revealed that oe‐Smurf1 treatment enhanced the expression of Smurf1, but the expression of KLF2 was declined in NSCs; oe‐KLF2 treatment did not influence the expression of Smurf1, but KLF2 expression was increased in NSCs. Transfection with oe‐Smurf1 + oe‐KLF2 increased the expression of Smurf1, whereas the expression of KLF2 was diminished in NSCs, compared with transfection with oe‐NC + oe‐KLF2 (Figure 5A,B). As demonstrated in Figure 5C‐E, the overexpression of Smurf1 decreased the viability and migration properties of NSC but increased NSC apoptosis, which was contrary after the overexpression of KLF2. Moreover, the effects of oe‐KLF2 on the viability, migration and apoptosis properties of NSC were nullified by additional treatment with oe‐Smurf1. Therefore, these results indicated that Smurf1 induced apoptosis but repressed the proliferation and migration abilities in NSCs by degrading KLF2.

### 3.6 KLF2 inhibited the expression of ATF2 to promote proliferation and migration but suppress apoptosis in NSCs

**FIGURE 6:**
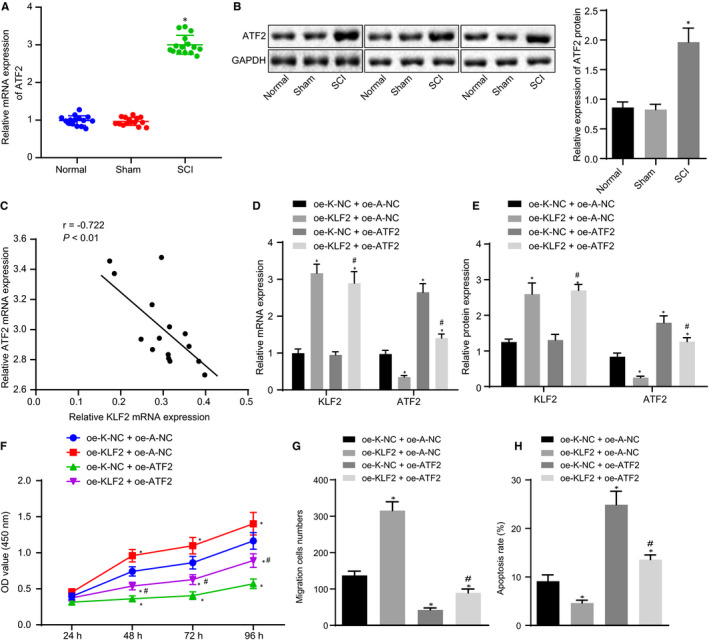
The overexpression of KLF2 down‐regulates ATF2 to induce proliferation and migration, but represses apoptosis in NSCs. A, The expression of ATF2 mRNA in the spinal cord of rats after modelling detected by RT‐qPCR normalized to GAPDH. B, The expression of ATF2 protein in the spinal cord of rats after modelling detected by Western blot analysis normalized to GAPDH. C, The correlation between the expressions of KLF2 and ATF in SCI rats analysed by Pearson's correlation analysis (n = 15). NSCs were transfected with oe‐K‐NC + oe‐A‐NC, oe‐KLF2 + oe‐A‐NC, oe‐K‐NC + oe‐ATF2 and oe‐KLF2 + oe‐ATF2. D, ATF2 and KLF2 mRNA expression in NSCs measured by RT‐qPCR normalized to GAPDH. E, Expressions of ATF2 and KLF2 protein in NSCs detected by Western blot analysis normalized to GAPDH. F, NSC viability measured by CCK‐8. G, NSC migration evaluated by Transwell assay. H, NSC apoptosis assessed by flow cytometry. ^*^ *P* < .05 vs normal rats and sham‐operated rats, NSCs transfected with oe‐K‐NC + oe‐A‐NC, ^#^ *P* < .05 vs NSCs transfected with oe‐K‐NC + oe‐ATF2. Comparisons among multiple groups were performed with one‐way analysis of variance (ANOVA), and cell viability at different time‐points was compared using two‐way ANOVA. Pearson's correlation analysis was conducted for the relationship between KLF2 and Smurf1. Rats: n = 15 in each group. The cell experiment was repeated three times

The downstream mechanism of KLF2 in SCI was investigated. ATF2 was found to be a transcription factor with the binding site on the KLF2 gene promoter, according to the GeneCards database. RT‐qPCR and Western blot analysis demonstrated that the expression of ATF2 in SCI rats was significantly higher than that in normal rats and sham‐operated rats (*P* < .05; Figure 6A,B). Pearson's correlation analysis showed that the KLF2 level was negatively correlated with the expression of ATF2 in SCI rats (15 rats) (*P* < .05; Figure 6C). To verify whether KLF2 could inhibit the expression of ATF2, KLF2 and ATF2 were overexpressed in NSCs cells, followed by conducting RT‐qPCR and Western blot analysis. Our results indicated that after up‐regulating KLF2, KLF2 expression was elevated; however, the expression of ATF2 was decreased in NSCs. Nevertheless, by overexpressing ATF2, the expression of KLF2 was not affected but the expression of ATF2 was up‐regulated in NSCs. In comparison with oe‐K‐NC + oe‐ATF2‐transfected NSCs, oe‐KLF2 + oe‐ATF2‐transfected NSCs exhibited an increase of KLF2 expression but a decrease of ATF2 expression (Figure 6D,E). CCK‐8 (Figure 6F), Transwell assay (Figure 6G) and flow cytometry (Figure 6H) were conducted to measure the viability, migration and apoptosis properties of NSC, respectively. The results revealed NSC viability and migration were promoted, but apoptosis was repressed in NSC by the up‐regulation of KLF2. Nonetheless, the overexpression of ATF2 resulted in a contradictory outcome. Moreover, the effects of ATF2 overexpression on the viability, migration and apoptosis properties of NSC were reversed by additional KLF2 up‐regulation. Hence, KLF2 up‐regulation suppressed apoptosis whereas proliferation and migration were promoted in NSCs by the down‐regulation of KLF2.

### 3.7 Smurf1 inhibits the recovery of neurological function after SCI in rats by promoting the degradation of KLF2 and up‐regulating ATF2

**FIGURE 7:**
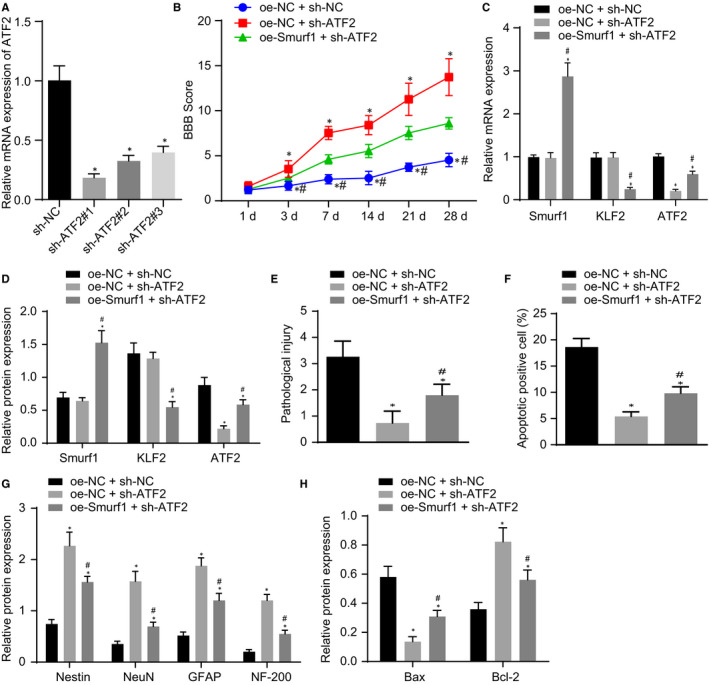
Smurf1 up‐regulation activates ATF2 by promoting KLF2 degradation to promote SCI and inhibit the recovery of neural function in SCI rats. A, Screening of the highest silencing efficacy of ATF2 by RT‐qPCR normalized to GAPDH. SCI rats were treated with oe‐NC + sh‐NC, oe‐NC + sh‐ATF2 and oe‐Smurf1 + sh‐ATF2. B, BBB score of SCI rats. C, RT‐qPCR detection of the mRNA expression of Smurf1, KLF2 and ATF2 in the injured spinal cord of rats normalized to GAPDH. D, Western blot analysis of the protein expression of Smurf1, KLF2 and ATF2 in the injured spinal cord of SCI rats normalized to GAPDH. E. HE staining analysis of the pathological changes of the spinal cord of rats. F. Apoptosis cells in the spinal cord of rats detected by TUNEL staining. G, Western blot analysis of protein expression of Nestin, NeuN, GFAP and NF‐200 normalized to GAPDH. H, The expression patterns of Bax and Bcl‐2 protein detected by Western blot analysis normalized to GAPDH. *P < .05 vs SCI rats treated with oe‐NC + sh‐NC, ^#^ *P* < .05 vs SCI rats treated with oe‐NC + sh‐ATF2. Comparisons among multiple groups were performed with one‐way analysis of variance (ANOVA) and data at different time‐points was compared using repeated‐measures ANOVA. Rats: n = 15 in each group

The aforementioned experiments showed that Smurf1 could promote the degradation of KLF2, whereas KLF2 decreased the expression of ATF2, speculating that Smurf1 could up‐regulate ATF2 by promoting the degradation of KLF2. To study the mechanism through which the Smurf1/KLF2/ATF2 axis affects SCI and neurological function recovery in vivo, we first performed RT‐qPCR to verify the silencing efficiency of ATF2. Our results revealed that sh‐ATF2#1 knockdown possessed the highest silencing efficiency of ATF2 (*P* < .05; Figure 7A). Thus, sh‐ATF2#1 was chosen for the subsequent experiments. Smurf1 was subsequently overexpressed, and ATF2 was silenced in SCI rats. Silencing ATF2 increased the BBB scores, which was reversed by the overexpression of Smurf1 (Figure 7B). RT‐qPCR and Western blot analysis indicated no significant difference observed in the expression of Smurf1 and KLF2, in comparison with that of the treatment with oe‐NC + sh‐NC; however, the expression of ATF2 was declined after treatment with oe‐NC + sh‐ATF2. Moreover, the treatment with oe‐Smurf1 + sh‐ATF2 elevated the expression of Smurf1 but reduced the expressions of KLF2 and ATF2 in SCI rats. In contrast to SCI rats treated with oe‐NC + sh‐ATF2, the expressions of Smurf1 and ATF2 in SCI rats treated with oe‐Smurf1 + sh‐ATF2 were significantly increased, whereas the expression of KLF2 was significantly decreased (Figure 7C,D). Furthermore, HE staining illustrated that the degree of pathological injury was diminished by the silencing of ATF2, which was rescued by the overexpression of Smurf1 (Figure 7E). TUNEL staining results revealed that decreased apoptotic cells were observed in SCI rats after silencing ATF2, which was negated by additional treatment with oe‐Smurf1 (Figure 7F). Additionally, Western blot analysis indicated that Nestin, NeuN, GFAP, NF‐200 and Bcl‐2 protein expression was noticeably increased whereas the expression of Bax protein was decreased in ATF2‐silenced SCI rats, which was nullified in the presence of additional of oe‐Smurf1 (Figure 7G,H). These results suggested that the overexpression of Smurf1 promoted KLF2 degradation to up‐regulate ATF2, thus promoting SCI and inhibiting the recovery of neural function in SCI rats.

### 3.8 Up‐regulation of miR‐125b promoted the recovery of neurological function in SCI rats via Smurf1/KLF2/ATF2 axis

**FIGURE 8:**
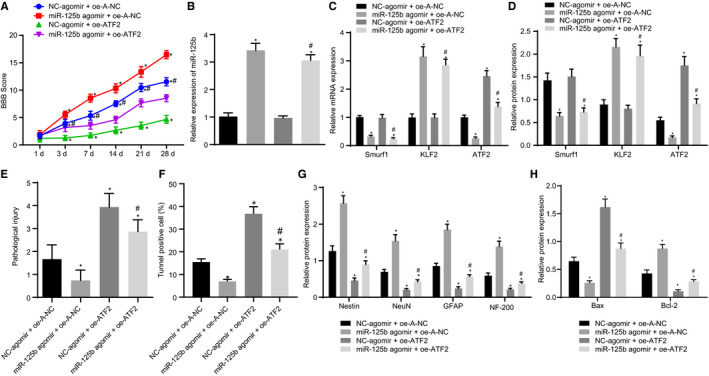
miR‐125b modulates the Smurf1/KLF2/ATF2 axis to repress SCI and induce the recovery of neural function in SCI rats. SCI rats were treated with NC‐agomir + oe‐A‐NC, miR‐125b agomir + oe‐A‐NC, NC‐agomir + oe‐ATF2 and miR‐125b agomir + oe‐ATF2. A, BBB score of SCI rats. B, RT‐qPCR detection of miR‐125b expression in the injured spinal cord of rats normalized to U6. C, RT‐qPCR detection of Smurf1, KLF2 and ATF2 mRNA expression in the injured spinal cord of rats normalized to GAPDH. D, Western blot analysis of Smurf1, KLF2 and ATF2 protein expression in the injured spinal cord of SCI rats normalized to GAPDH. E. HE staining analysis of the pathological changes of the spinal cord of rats. F. Apoptotic cells in the spinal cord of rats detected by TUNEL staining. G, Western blot analysis of protein expression of Nestin, NeuN, GFAP and NF‐200 normalized to GAPDH. H, The expression patterns of Bax and Bcl‐2 protein detected by Western blot analysis normalized to GAPDH. ^*^ *P* < .05 vs SCI rats treated with NC‐agomir + oe‐A‐NC, ^#^ *P* < .05 vs SCI rats treated with NC‐agomir + oe‐ATF2. Comparisons among multiple groups were performed with one‐way analysis of variance (ANOVA) and data at different time‐points was compared using repeated‐measures ANOVA. Rats: n = 15 in each group

We further aimed to investigate whether the up‐regulation of miR‐125b regulated the Smurf1/KLF2/ATF2 axis to inhibit SCI and promote the recovery of neural function in vivo. For this specific purpose, miR‐125b and ATF2 were overexpressed in SCI model rats. As shown in Figure 8A, miR‐125b agomir treatment significantly enhanced the BBB scores. However, the overexpression of ATF2 reduced BBB scores but its results were reversed by the addition of miR‐125b agomir. Our results of RT‐qPCR and Western blot analysis showed that the expression of miR‐125b and KLF2 in SCI rats treated with miR‐125b agomir + oe‐A‐NC was significantly enhanced compared with that of SCI rats treated with NC‐agomir + oe‐A‐NC, but the expression of Smurf1 and ATF2 was noticeably diminished (*P* < .05). On the contrary, no significant difference was observed in the levels of miR‐125b, Smurf1 and KLF2 in SCI rats treated with NC‐agomir + oe‐ATF2 (*P* > .05), whereas the expression of ATF2 was substantially elevated (*P* < .05); the expression of miR‐125b, KLF2 and ATF2 in SCI rats treated with miR‐125b agomir + oe‐ATF2 was markedly increased, whereas the expression of Smurf1 was decreased (*P* < .05). In comparison with SCI rats treated with NC‐agomir + oe‐ATF2, miR‐125b and KLF2 expression were increased whereas the expression of Smurf1 and ATF2 was decreased in SCI rats treated with miR‐125b agomir + oe‐ATF2 (*P* < .05; Figure 8B‐D). HE staining demonstrated that the degree of pathological injury was reduced by miR‐125b agomir but was elevated by oe‐ATF2, which was rescued by additional miR‐125b agomir (Figure 8E). Moreover, miR‐125b agomir‐treated SCI cells exhibited a reduction of apoptotic cells, whereas oe‐ATF2‐treated SCI rats showed an increased activity of cell apoptosis; however, the result was nullified by the additional treatment with miR‐125b agomir (Figure 8F). Besides, Western blot analysis documented that the expression of Nestin, NeuN, GFAP, NF‐200 and Bcl‐2 protein was noticeably enhanced but the expression of Bax protein was diminished observably in SCI rats after treatment with miR‐125b agomir. An opposite trend was observed in SCI rats after up‐regulating ATF2. The effect of oe‐ATF2 on the expression of Nestin, NeuN, GFAP, NF‐200, Bax and Bcl‐2 was reversed by additional treatment with miR‐125b agomir (Figure 8G,H). Collectively, the overexpression of miR‐125b mediated the Smurf1/KLF2/ATF2 axis, thus inhibiting SCI and promoting the recovery of neural function in SCI rats.

## 4 DISCUSSION

Spinal cord injury is characterized by neurological deficit, and the major focus of its cure is often emphasized on the regeneration of axons in the central nervous system.^20^ Recently, miRs have been reported to mediate various neurobiological processes, like cell proliferation, growth, differentiation and neural activity, along with the pathogenic processes of SCI, including apoptosis, demyelination, oxidation and inflammation.^21^ miR‐125b is also known to promote neurological recovery in rats with SCI.^10^ Although each miRNA is governed by many potential targets, it still remains a challenge to understand the roles of miR in SCI that depend on the identification of bona fide molecular targets. Based on the above, we intended to elucidate the mechanism by which the biological function of miR‐125b influences the neurological recovery after SCI.

Our data revealed that miR‐125b up‐regulated KLF2 by targeting Smurf1, thus repressing ATF2 and promoting neurological recovery in SCI rats. Initially, our study uncovered the down‐regulation of miR‐125b and KLF2, as well as the up‐regulation of Smurf1 and ATF2 in SCI rats. However, after the enforced expression of miR‐125b or KLF2 or silencing of Smurf1 or ATF2, neurological function recovery was promoted in SCI rats. It is also well‐known that miRs play a critical role in the progression of neurological disease by regulating neuronal communication in a tight balance.^7^ Mounting researches have demonstrated that miR‐125b promotes neurological function recovery in neurological diseases. For instance, a study conducted by Quiroz et al uncovered that miR‐125b promoted the neurological function recovery in SCI rats.^10^ In addition, miR‐125b mimic might prevent neuroinflammation and aberrant p53 network activation‐induced apoptosis during ischaemia‐reperfusion injury by down‐regulating TP53INP1.^22^ Also, miR‐125b plays a crucial role in orchestration of cell proliferation, differentiation and migration in neural stem/progenitor cells by targeting Nestin.^23^ Another research unravelled the depressive role of miR‐125b in cerebral ischaemia‐reperfusion injury by blocking Bax/Cytochrome C/Caspase‐3 apoptotic pathway.^8^ Additionally, another study elucidated that the up‐regulation of KLF2 was a promoter of neurological function recovery in rats with subarachnoid haemorrhage.^24^ In consent with our results, it has been documented that high expression of Smurf1 was observed after SCI in rats.^25^ The up‐regulation of ATF2 expression has also been reported in injured L5/6 dorsal root ganglia and spinal cord after nerve injury.^26^ Our results from the dual‐luciferase reporter gene assay depicted that Smurf1 was a putative target gene of miR‐125b. In consistent with previously reported data, our study confirmed that Smurf1 promotes the degradation of KLF2 via its E3 ubiquitin ligase function.^13^ It was also reported that KLF2 repressed the expression of ATF2,^15^ which was in line with our results.

Our results exhibited that overexpressing miR‐125b or KLF2 and silencing Smurf1 or ATF2 promoted the proliferation and migration in NSCs, but repressed the activity of apoptosis in NSCs, respectively. A recent study has shown that the overexpression of miR‐125b significantly promoted the differentiation and migration abilities of NSC.^23^ Of note, the up‐regulation of miR‐125b attenuated the activity of cardiomyocyte apoptosis induced by hypoxia in vitro; a decrease in activity of cardiomyocytes apoptosis was also confirmed in vivo after acute myocardial infarction.^27^ Furthermore, it has been demonstrated that the ectopic expression of Smurf1 could enhance the neuronal necroptosis to promote LPS‐induced neuroinflammation.^12^ Ling *et al* observed that the overexpression of KLF2 could promote pancreatic acinar cell proliferation, but cell apoptosis was repressed in acute pancreatitis following the overexpression of KLF2.^28^ Taken together, these reported findings speculated that miR‐125b had promoted proliferation and migration, whereas apoptosis was inhibited in NSCs via Smurf1/KLF2/ATF2 axis, thus alleviating SCI.

Our data indicated that miR‐125b served as a potential therapeutic target of SCI by specifically promoting neurological function recovery in SCI via Smurf1/KLF2/ATF2 axis. However, there are several limitations in this research. For instance, each miRNA could have numerous targets, and a target gene corresponds to different miRNAs. These findings may speculate that despite one or several miRNA‐regulated genes that can effectively modulate SCI, the development of a comprehensive therapeutic strategy of SCI still requires further investigation to evaluate the interaction among multiple genes regulated by miRNAs. In addition, more attention should be paid on the physiological and pathophysiological differences when associating the animal results to the human clinical setting. Further experiments on humans are warranted to determine the value of clinical application of miR‐125b in SCI. Finally, we used NSCs to establish the in vitro model. Nevertheless, the core mechanism of neurological dysfunction caused by damage is by the destruction of spinal cord neurons, which warrants further experiments about SCI on spinal cord neurons.

## CONFLICT OF INTEREST

The authors declare no competing interest.

## AUTHOR CONTRIBUTIONS

**Kunchi Zhao:** Data curation (equal); Funding acquisition (equal). **Ran Li:** Data curation (equal); Formal analysis (equal). **Qing Ruan:** Data curation (equal); Formal analysis (equal). **Chunyang Meng:** Data curation (equal). **Fei Yin:** Data curation (equal); Formal analysis (equal). **Qingsan Zhu:** Data curation (equal); Formal analysis (equal).

## Data Availability

The data sets generated/analysed during the current study are available.
